# Therapeutic Potential of *Prunus* Species in Gastrointestinal Oncology

**DOI:** 10.3390/cancers17060938

**Published:** 2025-03-10

**Authors:** Gabriela Mitea, Irina Mihaela Iancu, Verginica Schröder, Adrian Cosmin Roșca, Valeriu Iancu, Ruxandra-Mihaela Crețu, Horațiu Mireșan

**Affiliations:** 1 Department of Pharmacology, Faculty of Pharmacy, Ovidius University of Constanta, 900470 Constanta, Romania; gabriela.mitea@365.univ-ovidius.ro; 2 Department of Toxicology, Faculty of Pharmacy, Ovidius University of Constanta, 900470 Constanta, Romania; horatiu.miresan@univ-ovidius.ro; 3 Department of Cellular and Molecular Biology, Faculty of Pharmacy, Ovidius University of Constanta, 900470 Constanta, Romania; 4 Department of Analysis and Quality Control of Drugs, Faculty of Pharmacy, Ovidius University of Constanta, 900470 Constanta, Romania; cosmin.rosca@univ-ovidius.ro; 5 Department of Pharmaceutical Technology, Faculty of Pharmacy, Ovidius University of Constanta, 900470 Constanta, Romania; valeriu.iancu@univ-ovidius.ro; 6 National Institute of Research and Development for Biological Sciences, “Stejarul” Biological Research Centre, 060031 Bucharest, Romania; ruxycretu@yahoo.com

**Keywords:** oncology, *Prunus*, polyphenols, antineoplastic agents, apoptosis, colon cancer, colorectal cancer, gastric cancer

## Abstract

Background: Gastrointestinal tract cancers represent a significant worldwide health concern, accounting for almost one-third of cancer-related deaths. The existing chemotherapy drugs used in gastrointestinal cancers are ineffective, so prognosis is poor, recurrence and metastasis rates are high, and survival time remains short, necessitating the development of novel antitumor drugs that exhibit low toxicity and less potential for the development of drug resistance. This challenge is considerable, but evidence from the past decades supports the medicinal properties and functionalities of bioactive compounds such as flavonoids and acid phenolics with anticancer activities. Our purpose was to find data on the relationship between gastrointestinal cancer and bioactive compounds from *Prunus* species, focusing on their molecular mechanisms of action. Results: Studies highlight the potential of bioactive compounds from *Prunus* species to modulate the cancer cell signaling pathways involved in gastrointestinal tumorigenesis. Conclusions: The studies reviewed suggest that polyphenols from *Prunus* species exhibit promising gastrointestinal anticancer activities and could represent an adjunctive therapeutic strategy in cancer treatment. Further studies are necessary to validate these compounds’ therapeutic potential and their feasibility as cost-effective treatments for cancer.

## Simple Summary

Although gastrointestinal cancer management strategies have made great progress, gastrointestinal cancer remains an important cause of death worldwide. There is growing interest in exploring the potential of alternative cancer therapies that do not involve chemotherapy and radiotherapy, which are already known to produce negative health effects. Phytocompounds are increasingly acknowledged as promising agents for cancer therapy and prevention owing to their derivation from medicinal plants and their potential to combat cancer. This systematic review was undertaken to assess the effectiveness of bioactive compounds from *Prunus* species in treating gastrointestinal cancer either alone or in conjunction with other treatments.

## 1. Introduction

Cancer is a term describing a broad group of diseases, which are characterized by cell failure or the rapid and uncontrolled development of abnormal cells that can aggregate or spread throughout the body to form a mass or tumor, leading to uncontrolled growth elsewhere [1,2,3,4]. In recent years, it has emerged as the leading cause of death, with the global registry of cancer cases continuing to increase [1,5,6,7,8]; the number of cases is expected to rise steadily to 21 million by 2030 [1].

Cancer is a complex disease that is linked to several risk factors, such as chemical substances polluting the environment, genetic predisposition, hormonal disorders, oxidative stress, bacterial and viral infections, poor nutrition, and epigenetic disturbances [4,7,9,10,11]. These risk factors give rise to various forms of cancer, and, sometimes, therapeutics must be different even for the same type of cancer [4,7,9,10].

One of the major challenges with cancer cells is their capacity to evade apoptosis due to unpredictable mutations, leading to uncontrolled cell growth and the subsequent migration to different areas of the body [2,12,13]. Therefore, an ideal anticancer drug should specifically target cancer cells while sparing normal cells. This can be accomplished by reactivating the apoptotic mechanisms within cancer cells and effectively addressing multidrug resistance (MDR) [2,14].

In recent decades, the emergence of an increasing number of side effects of synthetic drugs has led to the use of medicinal plants as rich sources of clinically effective anticancer agents [15,16,17]. Approximately 60% of the anticancer drugs currently used are derived from plants, either novel phytochemicals or their secondary metabolites [9,18,19]. Today, there are a variety of natural cancer treatments that may be able to provide results comparable to chemotherapy and radiation therapy. They are much less toxic and invasive compared to standard methods of cytostatic treatment. Some of the most successful cancer treatment plans involve a combination of both natural and conventional therapies [20,21,22,23].

Medicinal plants containing bioactive compounds found in plant products (fruits, vegetables, and cereals) with pharmacological actions offer an alternative remedy for many diseases [5,24]. They are highly heterogeneous, encompassing many chemical classes, such as polyphenols, carotenoids, phytosterols, and tocopherols, which differ in their chemical structures (hydrophilic or lipophilic) [25,26,27]. Polyphenols are recognized for their application in the prophylaxis and therapy of many human cancers [6,28]. Polyphenols, which include flavonoids and catechins, have strong anticarcinogenic properties because their cellular interactions mechanisms are complex, they scavenge free radicals, repair deoxyribonucleic acid (DNA) damage, and inhibit inflammatory pathways like mitogen-activated protein kinase (MAPK) and nuclear factor kappa-light-chain-enhancer of activated B cells (NF-Kb). Furthermore, polyphenols inhibit the expression of the factors involved in extracellular matrix remodeling [6,29,30]. The interactions occurring between different phenolic categories also serve to increase their biological potential. Indeed, it was already documented that phenolics can pass through the cellular membrane and hence scavenge the radicals that can cause damage to cells and promote apoptosis [11,31]. However, research suggests that polyphenols can be used in combination with drugs to enhance their efficacy [32,33].

Additionally, it is well established that flavonoids exhibit anticarcinogenic activities, modifying different stages of cancer development, such as cell growth, angiogenesis, and apoptosis, but the exact signaling pathways for these activities are not known in detail [31,34]. Numerous flavonoids have been shown to be effective in reducing the incidence of major cancers such as breast, prostate, stomach, and colorectal cancer, demonstrating potential in the development of new drugs [11,34].

Based on the evidence that these compounds are abundant in medicinal herbs and dietary plants like tea, apples, berries, cocoa, and grapes [32,35,36], this review aims to emphasize the potential of *Prunus* species. The genus *Prunus* is widespread throughout the world, comprising about 430 species (cultivated, ornamentally, expanded nutritional potential) [11,37], renowned for their high polyphenolic content, which have demonstrated promising effectiveness in treating gastrointestinal cancers.

The focus on gastric, colon, and colorectal cancers is due to both the high prevalence of these cancers and the evidence from the published literature demonstrating that bioactive compounds from *Prunus* species may interfere with the signaling pathways involved in the development of these cancers. Importantly, these natural compounds have been thoroughly researched strategies for cancer treatment, and current studies continue to investigate their potential in combination with other compounds [11,34].

## 2. Materials and Methods

The literature search encompassed publications from 2000 to 2025, including PubMed, Web of Science, and Google Scholar. To provide the relevant information for this review, several keywords were used, such as “*Prunus* sp.”; “colon cancer”; “colorectal cancer”; “gastric cancer”, “polyphenols”; ”bioactive compounds”, “antineoplastic agents”; “apoptosis”; “in vitro”; “in vivo”. The articles included reviews, guidelines, monographs, and conference proceedings. The goal was to collect data on the connection between gastrointestinal cancer and the bioactive compounds (polyphenols, flavonoids, anthocyanins) present in *Prunus* species, with an emphasis on their molecular mechanisms of action. The most significant findings were compiled into tables and figures. Most of the research articles that fulfilled the eligibility criteria were conducted in vitro, while relatively few clinical research articles have reported in vivo studies.

## 3. The Global Context of Gastrointestinal Cancer

Gastrointestinal (GI) cancers include esophageal, stomach, and colorectal malignancies, representing some of the most common malignant tumors worldwide [38,39]. These cancers present in different clinical forms, although they arise from the same and related tissues [40]. Over the last century, despite advances in economic development, healthcare systems, and public health, GI cancers continue to present huge medical and economic challenges [41,42]. When it comes to genetic factors, the two most prevalent hereditary colorectal cancer (CRC) syndromes are Lynch syndrome (hereditary nonpolyposis CRC) and familial adenomatous polyposis (FAP), which together represent 5–10% of colorectal cancer cases [41,43].

Globally, gastric cancer (GC) is recognized as the third leading cause of death and the fifth most frequent malignancy among other cancers. With modern treatment techniques, the incidence and mortality rate of gastric cancer continues to increase, with the survival rate of GC worldwide averaging around 30% [8,10]. Colon cancer (CC) is among the top five cancers that are diagnosed among the sexes, causing around 600,000 deaths each year. In 2020, the number of reported cases of CC was approximately 1.9 million, and this number is projected to increase by 60% (~2.2 million) by the end of 2035 [44]. According to statistics, colorectal cancer accounted for 9.4% of all deaths globally in 2020. Regarding incidence, CC ranks third for men and second for women in the category of the most common cancers. In countries with medium or high levels of human development, the morbidity and mortality rates in the population under 65 have been increasing by 1% and 1.3% per year [45,46].

**Figure 1:**
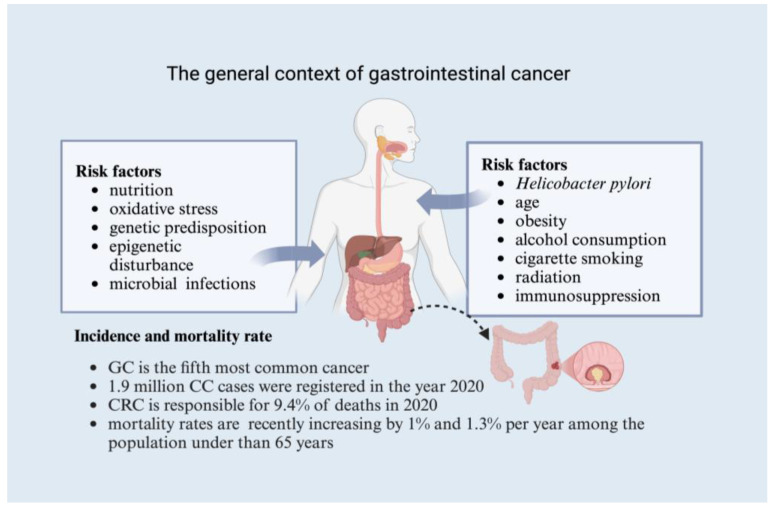
Risk factors, incidence, and mortality (GC—gastrointestinal cancer, CC—colon cancer, CRC—colorectal cancer) (created in https://BioRender.com), Schroder, V. (2025).

Gastric cancer is a very complex disease that can be influenced by environmental and genetic risk factors (Figure 1) [10,47]. The most important and widely studied risk factor associated with this pathology is *Helicobacter pylori* (*H. pylori*) infection, which usually varies significantly among different ethnic populations [10,48]. Eliminating *H. pylori* has been linked to a better prognosis [48]. There are many other factors that contribute to the increased death rate among gastric cancer patients, including age, diet low in fruits and vegetables, high salt intake, and intestinal metaplasia, which are considered independent risk factors [10,49,50].

In addition, it has been observed that, in most cases, colon cancer occurs sporadically and is associated with risk factors such as age, diet, race, inflammatory bowel disease, alcohol consumption, history of prior radiation, immunosuppression, cigarette smoking, and gene mutations (adenomatous polyposis coli (APC), deleted in colon cancer (DCC), K-ras, p53, BRAF) (Figure 1) [43,51]. Physical activity is widely recognized for its potential to lower cancer risk, and it has been firmly established as a risk factor for colon cancer, with evidence of its impact on rectal cancer being less conclusive [41,52].

Epidemiological studies and clinical research have emphasized a strong connection between the small intestine, inflammation, intestinal microbiota, and colon cancer [53].

The influence of environmental as well as host genetic factors lead to the multifactorial development of CRC. The APC gene was the first genetic mutation identified as being involved in the Wnt/β-catenin pathway and plays a role in regulating cell proliferation [54,55,56]. Sociopolitical factors that may increase the prevalence of CRC also include poverty and the level of education; in addition, ethnicity has been reported to influence the risk of CRC [57,58,59]. The risk of CRC is increased for patients who have already been diagnosed with a certain type of cancer, as well as those with a family history of cancer, inflammatory bowel disease (IBD), colon polyps, diabetes, or cholecystectomy [41,45,52].

Recent reports analyzing the microbiome of patients with CRC indicate that changes in the composition and structure of the microbiota, as well as the physiological functions of the normal gut flora, may promote or increase the progression of CRC [51,52,54].

The gut microbiome (or microbiota) is a complex population of living organisms: bacteria, viruses, fungi, and protozoa that inhabit the human gastrointestinal tract [52]. In normal health conditions, the metabolic activities of the intestinal microflora allow drugs to be converted into active compounds. This transformation takes place due to enzymatic activity that leads to the generation of compounds with low polarity and low molar mass that are delivered to a specific organ [52,60].

**Figure 2:**
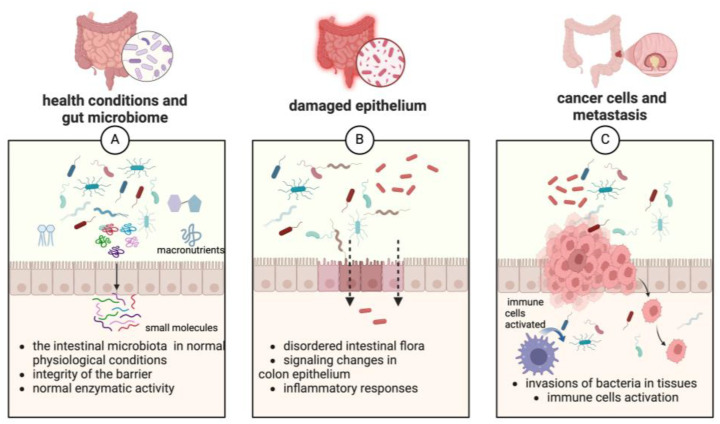
Changes in gut microflora and epithelial cell integrity in the process of cancer formation (created in https://BioRender.com), Schroder, V. (2025).

Normally, the gut microbiota functions to maintain gut barrier integrity (Figure 2), protect against pathogens, and modulate immune responses. However, its balance can be disrupted when beneficial bacteria decrease and epithelial cells undergo change, leading to DNA damage, alterations in cell cycles, activation of immune responses, and the deterioration of the intestinal barrier function. This disturbance leads to disordered intestinal flora in colorectal, liver, and lung cancer, and other malignancies (Figure 2) [52,60]. The transformation to a proinflammatory type of microbiota may be associated with ageing, which may lead to a decreased ability of the immune cells to suppress inflammation in the colon. Dysbiosis, inflammation, and reductions in the numbers of butyrate-producing bacteria associated with increased intracolonic pH contribute to CRC [54,61,62].

In recent research, an increased microbial biodiversity was identified in the CRC microenvironment, with altered abundances of commensal and pathogenic bacterial taxa such as *Fusobacterium*, *Providencia*, *Streptococcus bovis*, *Clostridium septicum*, *Enterococcus faecalis*, *Escherichia coli* strain NC101, *Peptostreptococcus anaerobius*, *Bacteroides fragilis,* and *Akkermansia mucinphila*, driving tumor formation and/or progression [54,63,64,65]. *Helicobacter pylori* infection triggers an inflammatory response, alters the gastric pH, disrupts the gastric microbiota composition, facilitates colonization by other bacteria, and initiates gastric cancer [60].

Studies have reported that the beneficial effects of therapies used against cancer may be influenced by the gut microbiota by modulating the response, efficacy, and toxicity of radiotherapy, chemotherapy, and immunotherapy [54]. Therefore, regulating the activities of intestinal microorganisms opens new possibilities for increasing the efficacy of tumor immunotherapy and overcoming immune resistance [66].

Among all the classification systems reviewed, the 2010 World Health Organization (WHO) classification is the most detailed. In addition to gastric adenocarcinomas, the WHO classification describes other less common gastric tumors. The classification of gastric adenocarcinomas also includes subtypes such as tubular, papillary, mucinous, and mixed carcinoma [47]. Symptoms occur concurrently and include abdominal pain, weight loss, nausea, vomiting, dysphagia, dyspepsia, fatigue, and depression [67].

The staging of colon cancer follows the TNM classification system (tumor/node/metastasis), in which stages are defined according to the characteristics of the primary tumor (T), the involvement of regional lymph nodes (N), and the extent of distant metastases (M). Furthermore, metastasis can be defined either clinically or pathologically, depending on whether it is assessed through preoperative clinical evaluations or pathological examinations of metastatic tissue [68]. Colon cancer originates in the bowel’s mucosa, growing into both the lumen and the bowel wall, and may spread to nearby organs. Symptoms are typically linked to larger tumors or more advanced stages of the disease and may not be exclusive to colon cancer.

The most frequently reported symptoms in patients with CC but also with CRC include fatigue, insomnia, rectal bleeding, an abdominal mass or abdominal pain, along with symptoms such as constipation or diarrhea, unexplained weight loss, or even iron-deficiency (anemia). There are other nonspecific symptoms that could also be conclusive, such as unexplained lack of appetite or deep vein thrombosis [45,52,69,70].

## 4. Key Signaling Pathways Associated with Gastrointestinal Cancer

**Figure 3:**
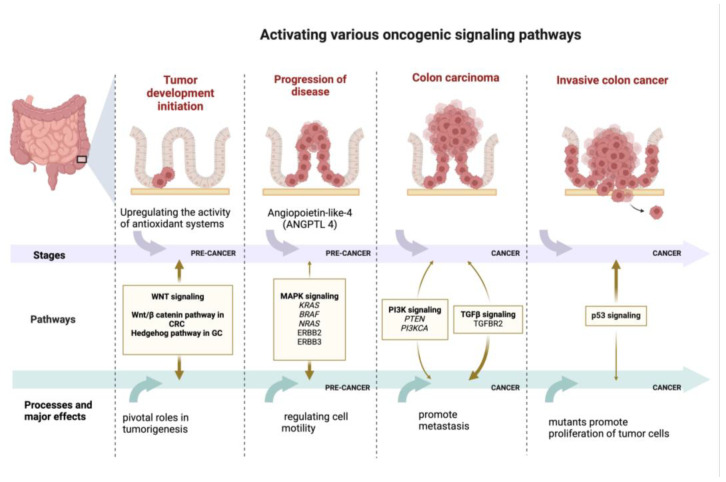
The oncogenic pathways, processes, and major effects in CC cancer (created in BioRender. Schroder, V. (2025) https://BioRender.com/i00x511). Wnt/β-catenin signaling pathway has an important function in regulating essential cellular processes like determining organ development during embryogenesis, motility, polarity, and stem cell renewal; it is known that one of the driving forces in cancer is the impairment of the main physiological signaling pathways present in tumor cells caused by the presence of certain mutations; the hedgehog pathway is a fundamental signaling pathway in organogenesis; ANGPTL4: angiopoietin-like 4, secreted protein involved in angiogenesis; MAPK: signaling pathway that plays a role in regulating cell motility; MAPK: mitogen-activated protein kinase; *KRAS* and *NRAS*: *RAS* family genes, Ras: small G-protein; *BRAF*: B-Raf proto-oncogene serine/threonine kinase; ERBB2 and ERBB3: ERBB epidermal growth factor receptor; TGFBR2: transforming growth factor beta receptor 2; *PIK3CA*: phosphoinositide-3-kinase, catalytic, alpha polypeptide genes, where mutations are presented in 10–30% of colorectal cancers; *PTEN* gene phosphatase acts as a tumor suppressor; p53 signaling: p53 protein is a transcription factor, where mutations promote the proliferation of tumor cells.

Most of the signaling pathways listed below play an essential role in the tumorigenesis (Figure 3), progression, and metastasis of gastric, colon, and colorectal cancers, but some of them have been shown to be cancer-specific, for example, the colorectal cancer-specific Wnt/β-catenin pathway and the gastric cancer-specific hedgehog (Hh) pathway.

- The phosphatidylinositol 3-kinase/protein kinase B/mechanistic target of rapamycin (PI3K/AKT/mTOR) signaling pathway is a highly relevant pathway for many pathological conditions, including cancer progression. It regulates the autophagy, apoptosis, and survival of various cancers, including malignant tumors of the gastrointestinal tract [10,71,72].
- NF-kB signaling pathway. Persistent inflammation is a well-known mechanism that may lead to the onset of neoplastic processes and may also stimulate tumorigenesis by inducing DNA damage. In addition, cytokine-specific receptor-mediated signaling pathways are modulated by inflammatory processes and control some of the most vital aspects of tumor initiation and promotion in CRC, such as activating signal transducer and activator of transcription 3 (STAT3) through interleukin-6 (IL-6) and interleukin-11 (IL-11) signaling as well as tumor necrosis factor (TNF) receptor-mediated and interleukin-1 (IL-1) receptor-mediated NF-κB activation [4,73,74,75,76,77,78]. These processes include promoting cell proliferation (by regulating cyclin D, c-Myc, and IL-6, which regulate growth-promoting signals), inhibiting apoptosis (by inhibiting apoptotic genes including B-cell lymphoma 2 (Bcl-2) and B-cell lymphoma extra-large (BclxL) transcription), promoting angiogenesis (inducing vascular endothelial growth factor (VEGF) expression), promoting tumor invasion (through E-selectin and matrix metalloproteinases (MMPs)), promoting epithelial–mesenchymal transition (EMT) and colon cancer stem cells (CSCs), and mediating tumor drug resistance [4,45,75,76,77,78,79,80]. It has been shown that the exposure of gastric epithelial cells to *H. pylori* infection causes the rapid activation of NF-κB, with the nuclear translocation of p50/RelA and p50/p50 dimers leading to potent messenger RNA (mRNA) accumulation for interleukin-8 (IL-8) in vitro [10].
- The Janus kinase/signal transducer and activator of transcription (JAK/STAT) signaling pathway is commonly activated by growth factors and cytokines, playing an important role in inflammation-driven colorectal cancer. It influences the tumor microenvironment (TME), angiogenesis, and the mechanisms that enable the cancer to evade immune system detection [4,73,74,75,76,77,78]. Studies using in vivo and in vitro models have demonstrated that JAK/STAT signaling is deregulated in malignant transformation and, therefore, may contribute significantly to the expansion of a variety of solid tumors and hematopoietic malignancies [81]. Specifically, in gastric tumor formation, the dysregulated activation of the JAK/STAT pathway has been implicated [10,81].
- The Wnt/β-catenin signaling pathway has an important function in regulating essential cellular processes like determining cell fate, adult homeostasis, organ development during embryogenesis, motility, polarity, and stem cell renewal. It is known that one of the driving forces in cancer is the impairment of the main physiological signaling pathways present in tumor cells caused by the presence of certain mutations [10,54,82]. Moreover, in a recent study, for the first time, it has been shown that the virulence factor FadA, from *Fusobacterium nucleatum*, interacts with E-cadherin, which is a cell surface molecule that mediates metastasis in CRC by activating an essential component of the Wnt/β-catenin signaling pathway, which is known to be the most damaged by mutations in CRC [63,83]
- Hippo signaling pathway. The crosstalk between Wnt and other pathways is significant for CRC pathogenesis. The synergism that possibly influences apoptosis and cell growth in CRC is expressed through the transcriptional regulation of Yes-associated protein (YAP), an effector of the Hippo pathway, by the β-catenin/T-cell factor 4 (TCF4) complex [4,73,74,75,76,77,78]. The dysregulation of Hippo pathway signaling in GC and other solid tumors contributes to unregulated cell division and the activation of metastasis [10].
- Notch signaling pathway. Notch can modulate the Wnt pathway signaling, demonstrating a complex relationship with Wnt. APC mutation disturbs Wnt signaling but activates Notch, a pathway that is important for colonic lesions early in tumorigenesis. Also, further interplay among the Wnt and Ras pathways causes APC mutations to stabilize Ras to enhance its oncogenic potential by modifying its proteasomal degradation [4,73,74,75,76,77,78]. Proliferation, tumor cell survival, and tumorigenesis in vivo are promoted by the activation of Notch signaling through several isoforms of hairy and enhancer of split 1 (HES1) found in different cellular contexts [10,82].
- Hh signaling pathway. Currently, Hh signaling is increasingly recognized for its putative oncogenic role in CRC pathology (Figure 3). It has emerged as a master regulator in cell proliferation, differentiation, and embryonic patterning [84]. The Hh family of proteins control numerous cellular processes in mammals—their roles include survival, apoptosis, proliferation, differentiation, invasion, and migration [4,73,74,75,76,77,78]. Hh signaling has been identified as a key factor in the formation and differentiation of gastric glands during embryonic development. In the adult stomach, the Hh pathway is a regulatory pathway that governs the differentiation of gastric epithelial cells and the maintenance of their maturation state, being indispensable for the physiology of the stomach. Gastric cancer cells exhibit both increased sonic hedgehog (SHH) expression and higher levels of Patched 1 (PTCH1) receptor. As a result, the overproduction of SHH activates Hh signaling, which in turn drives GC cell proliferation and progression [10,82].
- MAPK signaling pathway. Numerous studies have shown that the extracellular signal-regulated kinase (ERK)/MAPK pathways and downstream molecules (*KRAS* and *NRAS*: *RAS* family genes; Ras: small G-protein; *BRAF*: B-Raf proto-oncogene serine/threonine kinase; ERBB2 and ERBB3: ERBB epidermal growth factor receptor) [79,85,86] play a role in regulating cell motility in both gastric cancer (GC) and normal epithelial cells. Specifically, in GC, the ERK pathway modulates MMP activities, thereby influencing cell migration and tumor invasion. In addition, the angiopoietin protein-like-4 (ANGPTL4) induced following hypoxia exerts multiple influences on gastric scirrhous carcinoma neoplasia (Figure 3). Through the ANGPTL4-induced activation of the focal adhesion kinase (FAK)/Src/phosphoinositide 3-kinase (PI3K)-AKT/ERK signaling pathway, GC cells acquire anoikis resistance, which contributes to peritoneal metastasis [4,73,74,75,76,77,78].
- Transforming growth factor beta (TGF-β/Smad) signaling pathway. The TGF-b signaling pathway is an important modulator of intestinal homeostasis and inflammation; thus, the dysregulation of this pathway may be associated with carcinogenesis [87], related to the presence of inflammation in the gastrointestinal tract. In the early stages of neoplastic development, it acts as a tumor suppressor, but, in the later stages of the disease, it can shift its role to facilitate EMT and promote metastasis [4,10,73,74,75,76,77].
- TLRs signaling pathway. TLRs are type I transmembrane glycoproteins that exhibit a structure containing a repetitive sequence in the extracellular domain that is rich in leucine, a highly conserved homologous Toll/IL-1R domain (TIR) in the cytosolic region, and a transmembrane domain and a homologous Toll/IL-1R domain, with similarities with the signaling domain of IL-1R family members [10].

Reactive oxygen species (ROS) are known to initiate and progress diseases such as cancer [88]. They drive tumor-initiating processes through the activation of various oncogenic signaling pathways, DNA mutations, alteration of the tumor microenvironment, escape from immune surveillance, and establishment of metastasis and angiogenesis [2,89,90]. To overcome the harmful effects of ROS, cancer cells adapt to oxidative stress by upregulating the activities of antioxidant systems [2,4,88,91].

The loss of p53 protein plays a significant role in tumor progression through interactions with the transcription of other genes that are involved in advancing metastatic stages (Figure 3) [92].

In recent years, molecular signal pathways are increasingly attracting attention as sources of therapeutic targets for colon cancer [82,93]. As a result, focusing on the apoptosis pathway has become a key area in the development of potential anticancer therapies. The manipulation of apoptosis pathways in cancer cells is a rapidly advancing field, showing significant promise for creating new cancer treatments. In recent years, this approach has gained momentum, yielding encouraging results [12,94]. Several natural compounds have shown the ability to influence signaling pathways and regulate the expression of the genes involved in cell cycle control, differentiation, and apoptosis [19,80].

## 5. Therapeutic Strategies

Conventional therapeutic strategy approaches for GC, CC, and CRC are based on endoscopic and surgical resection, preoperative and palliative chemotherapy, biologic therapy, local radiotherapy, targeted therapy, and immunotherapy [45], which are used by oncologists to cure these conditions [53,60,95,96].

In advanced gastric cancer stages, treatment starts with a platinum and fluoropyrimidine doublet as the first line. Targeted therapies include trastuzumab, ramucirumab, and nivolumab or pembrolizumab. For patients with metastatic disease, the standard first-line treatment approach is a combination of 5-fluorouracil (5FU)/platinum chemotherapy along with trastuzumab for tumors that are positive for human epidermal growth factor receptor 2 (HER2). In the second-line setting, most patients receive ramucirumab + paclitaxel, but those that are microsatellite instability high (MSI-H) receive pembrolizumab [49,97].

The current chemotherapy treatments include single-agent therapies, primarily fluoropyrimidine (5-FU), as well as combination regimens incorporating oxaliplatin (OX), capecitabine (CAP, also known as XELODA or XEL) and irinotecan (IRI). The most common combination therapies in first-line treatment are CAPIRI (CAP + IRI), FOXFIRI (5-FU + IRI), FOLFOX (5-FU + OX), and XELOX or CAPOX (CAP + OX). For patients with poor performance or low risk of deterioration, single-agent therapy is recommended. Additional agents appear to have similar efficacy, with differences primarily in side effects [43,96,98].

For treating CRC, the most common chemotherapeutic agents are platinum derivatives (OX), antimetabolites (CAP, 5 FU, topoisomerase inhibitors (IRIs), and tegafur/uracil (UFT) [80,99]. Molecular-targeted drugs include cetuximab, ramucirumab, binimetinib, bevacizumab, encorafenib, aflibercept beta, panitumumab, regorafenib hydrate, and the immune checkpoint inhibitor pembrolizumab [100,101]. Treatment approaches also involve combining targeted therapies with traditional chemotherapy, such as the use of cetuximab alongside folinic acid, 5-fluorouracil, and either oxaliplatin or irinotecan [53,99].

Multidisciplinary team management and shared decision making are recommended. Qualifying statements with further details related to the implementation of guideline recommendations are also included [102,103].

## 6. The Role of Exploring Natural Therapies in Cancer

Medicinal plants constitute about 85% of the resources utilized in traditional medicine, which is based on the use of plant products [5]. It is also known that secondary bioactive plant metabolites have therapeutic properties [5,104]. The production of these secondary metabolites can be altered by the specific environmental stress on each plant species or by specific developmental periods, resulting in a dynamic phytochemical profile unique to that species [80,105]. Such diversity within nature represents an extensive source for possible beneficial compounds for human use [104].

According to the literature, over 3000 plants globally have been identified for their anticancer properties [1,106]. Many of the bioactive substances found in these natural products have anti-inflammatory, antioxidant, and antiproliferative qualities, which in turn make them desirable candidates for cancer biology interventions [107]. They exert their anticarcinogenic effects by interfering with tumor induction, growth, and progression through different mechanisms [29]. Many compounds from medicinal or dietary plants have been reported as chemopreventive agents capable of inhibiting DNA damage and retarding or reversing carcinogenesis in in vitro and in vivo bioassays [3]. The identification of new sources of phytochemicals is an essential step in improving the translation of these laboratory findings to clinically efficient drugs [108]. The use of natural-product-based chemotherapy combination therapies has become common in cancer treatment, aiming [1,106] to inhibit the growth of cancer cells, enhance efficacy, reduce dosages, prevent the development of drug resistance, and minimize the frequently observed side effects of chemotherapy [1,2,106,109,110]. Tumor immunotherapy may also produce enhanced effects by altering the gut microbiota, helping to regulate the immune response to tumors [66]. These biocompounds modulate a series of cellular mechanisms that become favorable to the efficiency of and reduction in therapeutic doses. All these benefits are associated with a decrease in the adverse effects of chemotherapy and an improvement in the patient’s general condition.

### 6.1. Natural Bioactive Compounds Used in Cancer Prevention and Therapy

In general, an ideal bioactive compound should be capable of inhibiting cell proliferation by halting the cell cycle, activating cancer-related signaling pathways, and triggering the caspase cascade to induce apoptosis. It may also possess the ability to reduce mitochondrial potential and enhance the expression of apoptotic proteins [5,77]. The anticancer properties of plants are linked to their ability to inhibit cancer-promoting enzymes, repair DNA, enhance the production of antitumor enzymes in cells, boost immune function, and trigger antioxidant effects [2].

The consumption of anti-inflammatory or antioxidant-rich foods is part of a cure for pathophysiology involving inflammatory processes or high levels of reactive oxygen species [91].

Phenolic compounds, categorized as secondary metabolites in plants and considered an important class of natural antioxidant substances [111], are categorized into alkaloids, terpenoids, polyphenols, and flavonoids [112]. Also, polyphenols can be classified into various categories based on the number of phenolic rings and the structural arrangements connecting these rings. Approximately one-third of the polyphenolic compounds found in food are phenolic acids, which are primarily divided into two main groups: hydroxycinnamic acid derivatives (such as caffeic acid, coumaric acid, ferulic acid, and sinapic acid) and hydroxybenzoic acid derivatives (including protocatechuic acid, gallic acid, and p-hydroxybenzoic acid) [7,112]. The wide variety in their structure accounts for their broad pharmacological effects, including anti-inflammatory, antibiotic, antiseptic, antioxidant, and antiallergic activities [111].

The long-term consumption of polyphenols has been linked to beneficial effects in the prevention of cancer, type 2 diabetes, as well as cardiovascular and neurodegenerative diseases [113].

Given that they are limited by problems related to absorption, bioaccessibility, solubility, microbial metabolism, digestion, and excretion, the low bioavailability of polyphenol has been a concern [113]. Recently, it has been suggested that polyphenol-rich compounds play a crucial role particularly in preventing colorectal cancer and enhancing sensitivity to radiation and chemotherapy [53], but there are uncertainties about the side effects of polyphenol supplements [114]. They can reduce the body’s ability to absorb iron, thiamine, or folic acid.

It is accepted that the daily intake of phenol-rich sources is an effective way to suppress these effects, due to their role in acting as antioxidant species by chelating or inhibiting metal ions during the initiation of free radical formation, thereby suppressing radical species [115]. Also, cyclooxygenase, lipoxygenases, and phospholipase A2 interact with the proinflammatory nuclear factor B, decrease the expression of inducible nitric oxide synthase (iNOS), and thus promote good health [11]. These abilities are correlated with their chemical specificity, referring to carboxyl, hydroxyl, and methoxy groups, which increase the cytotoxic effects on cancer cells, inhibiting tumor development and/or progression [11,29].

Significantly, research has noted the capacity of phenolics to play a huge role in anticancer effects, where antioxidant compounds have shown the ability to inhibit the PI3K/Akt pathway, which downregulates myeloid cell leukemia-1 (MCL-1) activity and thus results in the inhibition of antiapoptotic factors and the potentiating of prosurvival effects. In addition, antioxidants are also able to specifically bind to a certain cancer cell and then inhibit the specific kinase that is responsible for the regulation of the signaling pathway that results in resistance to chemotherapeutic drugs. This implies that antioxidants do not only work individually against cancer; they can also help to improve chemotherapeutic drugs and reduce their side effects on healthy non-malignant cells [29,37,114]. These compounds induce remarkable effects on human cancers by reducing the expression of a transcription factor regulating the expression of cytoprotective genes, reducing p53 activation, decreasing Bcl-2 expression and mitochondrial membrane potential, suppressing the expression of hypoxia-inducible factor 1 (HIF-1), and increasing cellular apoptosis with the downregulation of p-Akt expression [7]. Previous research has demonstrated that flavonoids have anticancer properties by inhibiting crucial enzymes involved in carcinogenesis. They are also found to influence the expression of genes related to cell cycle regulation, apoptosis, and angiogenesis in cancer cells [77,116].

Flavonoids, amongst the most important metabolites or bioactive compounds found in medicinal plants, exhibit a broad spectrum of therapeutic properties [5]. Other types of secondary metabolites found in plants are anthocyanins, flavanones, flavones, flavanols, and isoflavonoids [1,76]. Flavonoids exhibit various beneficial properties such as antioxidant, anti-inflammatory, and antibacterial effects [117], along with analgesic, antiallergic, antiviral, hepatoprotective, estrogenic, and antiestrogenic effects. They also exhibit cytostatic and apoptotic characteristics, making them good candidates for chemoprevention in cancer [77,104,111], including oral, rectal, and prostate cancer [1,77].

Anthocyanins are a subcategory of flavonoids found mainly in fruits; are responsible for imprinting the red, blue, and purple colors of flowering plants [115]; and protect against neurological and cardiovascular pathologies, diabetes, and cancers [111]. Anthocyanins are recognized for their antioxidant capacity and have demonstrated antitumor effects against colon and breast cancer. Anthocyanin metabolites synthesized by gut microbes may have controlling effects in mediating anticancer activity [66]. Along with the beneficial effects of anthocyanins against cancer and cardiovascular disorders, antioxidant, anti-inflammatory, and antiproliferative activities have been reported to affect cellular signaling pathways, cause cell cycle arrest, and activate apoptotic redox-sensitive transcription factors [115].

Pectin is one of the well-known soluble fibers that has been shown to restore the balance of the intestinal microbiota, inhibit the growth of tumor cells, and play an important role in slowing the progression of CRC [66].

### 6.2. Phytochemical Composition and Therapeutic Potential of Prunus Species

The family *Rosaceae* is a remarkable taxon found predominantly in the northern hemisphere and comprises more than 100 genera and 3000 species. For example, cherry, apricot, almond, peach, apple, pear, plum, hawthorn, strawberry, blackberry, and rosehip belong to *Rosaceae* [108]. The various biological and pharmacological effects of *Prunus* species may be beneficial in the treatment of a variety of diseases, including cancer. Studies carried out on different species of *Prunus* indicate the varied applications of extracts as nutraceuticals given their phytochemical profiles with valuable therapeutic potential, as shown in Table 1 [11,29,37,48,53,82,108,111,112,118,119,120,121].

**Figure 4:**
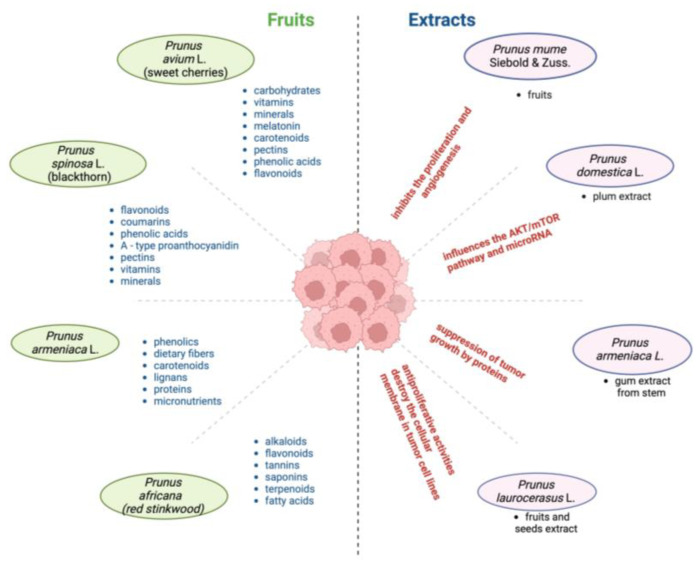
Bioactive composition of fruits and anticancer applications of *Prunus* sp. extracts.

Among these species, *Prunus avium* L. and its fruits (sweet cherries) have been extensively studied [11,118]. The well-documented biological effects of sweet cherry extract include its antioxidant and anti-inflammatory properties [62]. While sweet cherries are mainly composed of water, they are also packed with a wide range of nutrients, including carbohydrates (sugars and fiber), fatty and organic acids, amino acids, vitamins, minerals, and phytochemicals such as melatonin, carotenoids, pectin, phenolic acids (hydroxycinnamic derivatives), and flavonoids (anthocyanins, flavanols, and flavan-3-ols) (Figure 4) [118].

*Prunus spinosa* L. fruits, known as blackthorn, have antioxidant and antibacterial characteristics (Figure 4) and are used as astringents, diuretics, and purgatives. They are an abundant source of various compounds, including phenolic compounds such as flavonoids, coumarins, phenolic acids, and A-type proanthocyanidins, as well as pectin, vitamins, minerals, and organic acids [115,122].

*Prunus laurocerasus* L. (synonym *Laurocerasus officinalis*) fruit derives its nutritional and medicinal value from compounds such as vanillic, caffeic, chlorogenic, and benzoic acids, along with fructose, glucose, mannitol, ascorbic acid, anthocyanins, and tannins [48].

In Turkey, the fruits and seeds of *Prunus laurocerasus* L. are traditionally used in folk medicine to treat a variety of conditions, including kidney stones, stomach ulcers, and bronchitis as well as to strengthen bones. The seeds are utilized to help maintain the blood’s acid–base balance, while the fruits are used as treatments for eczema, hemorrhoids, and serve as a diuretic, antispasmodic, and antitussive [48]. *Prunus laurocerasus* L. has antiproliferative activities by destroying the cellular membrane in tumor cell lines (cervix, colorectal carcinoma, brain tumor) [108].

Different parts of *Prunus armeniaca* L. (apricot tree) are recognized for their anti-inflammatory, antipyretic, hepatoprotective, antiparasitic, and anticancer properties, being used in traditional medicine to treat a variety of digestive, respiratory, and gynecological conditions [119]. The fruits contain bioactive compounds rich in phenolics, dietary fibers, carotenoids, lignans, proteins, sugars, micronutrients, and fatty acids (Figure 4). These components have the potential to activate various anticancer processes and signaling pathways, including the activation of tumor suppressor proteins that help reduce the proliferation of cancer cells [112].

*Prunus africana* (Hook.f.) Kalkman is the most frequently utilized, which may be due to its high concentrations of chemical components that have anticancer properties, such as alkaloids, flavonoids, tannins, saponins, terpenoids, and fatty acids. Through apoptosis, antiproliferation activity, limiting replicative immortality, and antiangiogenesis effects, these phytochemicals demonstrate anticancer action [29]. It is reported to exhibit anti-inflammatory, analgesic, antimicrobial, antioxidant, antiviral, antimutagenic, antiasthmatic, and antiandrogenic activities, amongst others [120].

Kaempferol, extracted from *Prunus mume* Siebold and Zucc., is a flavonoid compound that has attracted widespread attention because of its anticancer, anti-inflammatory, antioxidant, antibacterial, antiviral, and other effects [80].

The almond *Prunus dulcis* (Mill) D.A. Webb is native to Mediterranean countries and other hot climates regions [82]. Almond seeds and oil have anti-inflammatory, immunostimulant, and antiproliferative effects and reduce irritable bowel syndrome symptoms (IBS), and they are also useful for treating constipation [82]. Almond seeds contain fixed oil, phenolic compounds, as well as some micronutrients, vitamins, and minerals, and they have different biological activities [82].

Plum (*Prunus domestica* L.) fruits are grown in many regions; the leading countries are the U.S.A, China, and Romania [11,37]. The abundant bioactive compounds of plum are phenolic acids, flavonoids, carotenoids, minerals, anthocyanins, and pectins [123]. Plums’ anticancer effects are associated with their antioxidant capacity; the antioxidants can either directly affect the cancer cell, or they can synergistically help in anticancer drug treatment [37]. Plums are known for their laxative and antioxidant activities, which further help in the prevention of cancers, particularly colon cancer [124].

## 7. Mechanisms of Action of the Major Classes of Bioactive Compounds Found in *Prunus* Species Against Gastrointestinal Cancer

In one study, *Prunus domestica* L. (plum) extract was found to significantly reduce cell viability and proliferation in colon cancer, with the effect being dose-dependent. Additionally, dried plum has been shown to lower the risk of colon cancer by reducing inflammatory markers, such as fecal bile acid concentrations, cecal microbial enzyme activities, and cecal oxygen radical absorbance capacity. Plum extract also influences the AKT/mTOR pathway and microRNA (miR-143), which are involved in cancer growth, thus suggesting chemopreventive potential for colon cancer. In addition, plum extract was also shown to decrease the expression of proinflammatory markers NF-κB (p65), vascular cell adhesion molecule-1 (VCAM-1), Cox-2, and iNOS [37].

Another study confirmed that *Prunus mume* Siebold and Zucc. has anti-inflammatory and antioxidant properties, which may have beneficial effects on the gastrointestinal tract by regulating intestinal secretion and gastrointestinal movements. *Prunus mume* Siebold and Zucc. suppresses the growth of endothelial cells (Figure 4) in CRC, inhibiting the expression of RelA, Bcl2, caspase 3, and cyclin D1 (CCND1) and promoting apoptosis-related proteins Bcl-2-associated X (Bax), cleaved caspase 3, and EGFR [45].

Prunetrin (prunetin 4-O-glucoside), a glycosyloxy isoflavone derived from *Prunus* species, interacts with receptor-interacting serine/threonine-protein kinase 3 (RIPK3) and triggers some cells death stages in gastric cancer [48]. Additionally, the glycosidic form of prunetin, known as prunetinoside, demonstrated a favorable effect specific to GC [75]. A recent study on prunetinoside, a flavonoid derived from *Prunus* sp., revealed key targets in gastric cancer (heat shock protein 90 (HSP90), cyclin-dependent kinase 2 (CDK2), and MMP1), and cell docking analysis confirmed the potential of the molecule to bind to these targets [5].

Recent investigations have demonstrated the anticancer activity of the active compounds found in bitter almond (*Prunus amygdalus* L. var. *amara*) on a diverse range of cell lines, including cervical, breast, lung, and colon cell lines. The results showed that apoptosis was induced and Ki-67 expression decreased with a dose-dependent and cell-line-type correlation [82,105].

Gum extracted from the stem of *Prunus armeniaca* L. was used to treat cancer. Bioactive compounds may exert anticancer properties through the stimulation of several anticancer processes and signaling pathways such as the suppression of tumor growth by proteins that suppress tumor cell proliferation (Figure 4) [112].

The relationship between the regular consumption of flavonoids and the initiation of carcinogenesis may serve as important evidence in determining their role in the gut microbiota as well as in tumor progression [53,60].

Several factors, such as molecular weight, glycosylation, and esterification, influence flavonoid bioavailability, creating uncertainty about their absorption in the human body. Flavonoids are metabolized in the small intestine, where their resulting metabolites are considered xenobiotics and are eliminated from the bloodstream. Unabsorbed compounds move to the colon, where they are altered by the colonic microflora. These catabolites can then enter the bloodstream and are eventually excreted in the urine. Additionally, flavonoids can influence the gut microbiota, promoting beneficial bacteria like *Bifidobacterium* and *Lactobacillus,* while having significant anti-gastrointestinal cancer effects [60,125].

Further research is needed to investigate the signaling and metabolism pathways that can highlight the positive effect of natural products on inflammatory and redox processes, thereby optimizing the potential of the bioactive compounds in nature [91].

## 8. Preclinical Studies on Anticancer Implications of *Prunus* Species

In vitro and in vivo analyses of *Prunus* extracts are vital to determine the possibility of their clinical application and for their commercialization as alternative strategies for cancer treatment [6,126]. As shown in Table 2, recent studies have demonstrated the in vivo and in vitro anticancer potential of plants and wild fruits [121].

**Figure 5:**
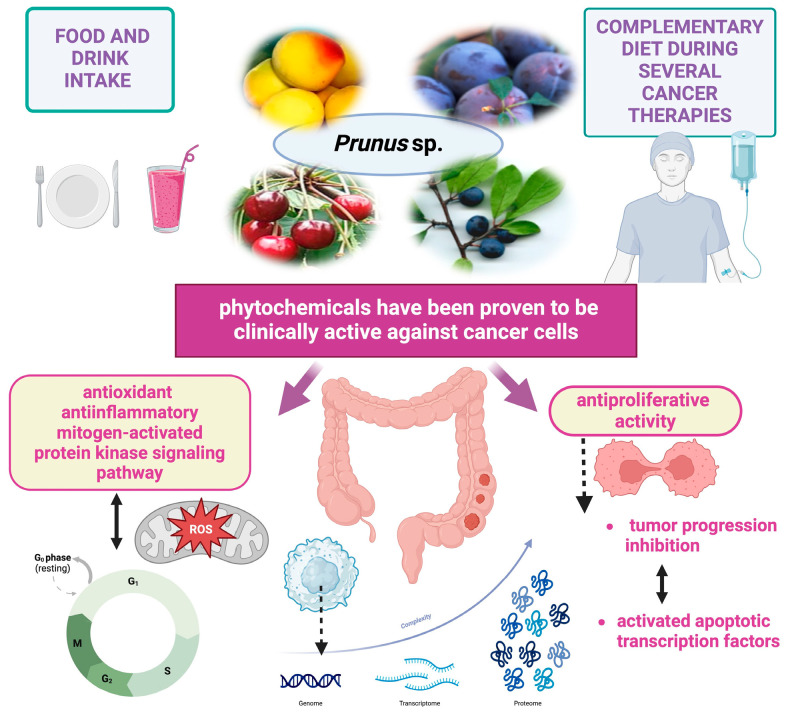
Design of the nutraceutical components of *Prunus* species and clinical application in current research (created in https://BioRender.com), Schroder, V. (2025).

A recent study demonstrated that polyphenols are primarily responsible for the antiproliferative and proapoptotic effects of sweet cherries’ bioactive compounds. In Caco-2 cells, the antioxidant properties of sweet cherries (*Prunus avium* L.) were linked to their anthocyanin content [11,118]. The phenolic compounds in sweet cherry extracts are absorbed by Caco-2 cells, where they restore the reduced glutathione/oxidized glutathione (GSH/GSSG) ratio, triggering an intracellular antioxidant response (Figure 5). Additionally, sweet cherry extract was found to affect the p38 mitogen-activated protein kinase (p38-MAPK) signaling pathway (Figure 5) [118].

According one study, in both animal and cellular experiments, kaempferol, extracted from *Prunus mume* Siebold and Zucc., significantly inhibited the growth, migration, and invasion of CRC cells through the RelA/NF-kB signaling pathway, as determined by network pharmacology and molecular docking. Moreover, kaempferol can regenerate chemosensitivity in 5-FU-resistant LS174-R cells, making it a potentially effective therapy for CRC [80]. Other authors found that MK615, a natural extract derived from the fruits of *Prunus mume* Siebold and Zucc., exhibited antiproliferative activity in vitro on human colon cancer cell lines SW480, COLO, and WiDr [127].

While additional research is required, the natural compounds in MK615 seem to demonstrate antineoplastic effects by triggering autophagy-related programmed cell death (PCD) in colon cancer cells [128]. Another similar study was conducted through experiments in mice and revealed that *Prunus mume* Siebold and Zucc. improved the symptoms of CRC model mice and participated in regulating the expression of RelA and apoptosis-related proteins [45].

Biocompounds from *Prunus spinosa* drupes blended with a nutraceutical activator complex (NAC), known as Trigno M complex, showed antitumor effects in both in vitro (HCT116 cell line) and in vivo (colon cancer xenografts in mice) models of colorectal cancer. Trigno M has been observed to inhibit 35% of HCT116 cell growth and colony formation, compared to the 80% inhibition seen with 5-fluorouracil. Tumor growth and morphological measurements in the 3D spheroid model were significantly decreased. In immunodeficient mice, the administration of Trigno M prevented tumor progression while reducing tumor necrosis [53].

A specific study was conducted based on the proanthocyanidins obtained from a *Prunus Spinosa* L. extract and tested in GLC and COLO320 cell lines, and a cytotoxic effect of proanthocyanidins on the cancer cell line was observed. It has been shown that ethanol and water extracts from *Prunus spinosa* L. fruits exhibit antitumor effects on a colorectal cancer cell line (HT-29) [115].

Although some preclinical studies suggest the potential of compounds from *Prunus* species (Figure 5) to inhibit cancer cell growth [11,45,53,115,119], these findings have not yet been translated into anticancer dietary supplements on the market.

One study demonstrated that almond oil is an effective antiproliferative agent against both Colo-320 and Colo-741 cells. The almond oils tested had similar effects in primary and metastatic colon carcinoma cells. Especially, animal studies further supported these findings, demonstrating that almond oil is associated with a reduction in the incidence of colon cancer [82].

In an extensive study, the methanolic extract of black splendor plum (*Prunus domestica* L.) was tested on Colon-26 adenocarcinoma cells (CRL-2638) as well as SW1116, HT29, and Caco-2 human colon cancer cells. After 72 h of incubation, protein concentration analysis from the cells showed that both the black amber and black splendor plum extracts had significant growth-inhibitory effects on the Caco-2 and NCM460 cells. Additionally, the plum extract (PE60) notably reduced the proliferation of Colon-26 cells [37].

In a comparative study, aqueous, ethanolic, and methanolic extracts from *Prunus armeniaca* L. kernels inhibited the growth of HCT-116 colon cells in a dose-dependent manner, with IC50 values of 33.6 and 36.3 µg/mL [119].

A preclinical study evaluated the potential of using *Prunus laurocerasus* L. fruit methanol extract against gastric cancer cell lines. The results revealed that 5 to 10 mg/mL concentrations of the extracts induced highly significant cell death in AGS and MKN-45 cell lines whilst preserving healthier human fibroblasts [48]. These findings indicate that *Prunus laurocerasus* L. fruit extracts have anticancer effects against gastric cancer, and, when further studied, the active components can be an alternative or adjuvant to standard chemical drugs used in the clinics [48].

## 9. Conclusions

Gastrointestinal cancer has become a global problem, with a negative impact on patients, their families, and the whole of society. Even though chemotherapeutic regimens and radiation therapy are more effective methods for treating cancer, they are nonselective, have substantial side effects, and can harm normal healthy tissues. It is necessary to develop a therapy based on efficacy that is safe and with few side effects after administration. Traditional medicinal plant research is leading to the development of novel bioactive compounds through approaches that combine ethnobotanical knowledge with modern scientific research. However, despite increased research efforts, existing information is insufficient for most of the dietary sources of polyphenols, hence the growing trend in the consumption of dietary supplements derived from these compounds, which increases the need for accurate and up-to-date information about their chemical and bioactive properties. Consequently, conducting more animal or human studies would be valuable to confirm whether the results from these in vitro studies can be applied more broadly. Additional clinical and in vivo research is needed to validate plums as functional foods for cancer treatment and prevention.

In addition, our study highlights the advantages of some extracts that show potential for anticancer therapy, and we propose that research on these species should be accelerated, considering several arguments with bioeconomy support. The species of the genus *Prunus* are accessible for research; the products used are easy to procure quickly and economically. The harvesting and storing of fruits with potential for medical use do not require special conditions or advanced technologies.

The products (fruits/seeds) are highly accessible, without affecting the environment or the natural stock of the plant, which happens with herbaceous medicinal plants whose products are limited (rhizomes, seeds, grass). The production and quality of the products can be predicted depending on the species used, the geographic area, and the cultivation area.

These medical products have an additional use as food: being a source of bioactive compounds provides an advantage for the development of micro-industries or production centers. By-products can be obtained, and added value can be given to the products.

## Figures and Tables

**Table 1:** Phytochemical composition and therapeutic potential of several *Prunus* species.

*Prunus* Species	Phytochemical Composition	Therapeutic Potential	Applications	References
*Prunus* *avium* L.	Water (>80%), carbohydrates (≈16%), fat (0.2%), melatonin (≈1586 ng/100 g), anthocyanins (1734 mg/100 g), other flavonoids (396 mg/100 g), phenolic acids (162 mg/100 g)	Antioxidant, anti-inflammatory	Reducing oxidative stress, inflammation, and potentially cancer-related signaling pathways	[11,118]
*Prunus* *spinosa* L.	Flavone/ols compounds (64.62 ± 0.58 mg/100 g of dry weight), phenolic acid compounds (38.36 ± 0.19 mg/100 g), and anthocyanins (0.63 µg/100 g)	Antioxidant, antibacterial, astringent, diuretic	Treatment of gastrointestinal issues, diuretic and purgative properties	[53]
*Prunus laurocerasus* L.	Phenolic acids (vanillic acid, caffeic acid, chlorogenic acid, gallic acid (GAE)), flavonoids (quercetin (QE), anthocyanins, tannins), and cyanogenic glycosides Total flavonoid content mgQE/100 g extract: 502.10 ± 6.85 mg QE/100 g Total phenolic content mg GAE/100 g extract: 461.31 ± 4.98 mg GAE/100 g	Antioxidant, protective against gastric cancer, antiproliferative, antispasmodic, diuretic, antitussive	Treatment of kidney stones, stomach ulcers, bronchitis, and eczemas; antiproliferative on tumor cells	[48,108]
*Prunus armeniaca* L.	Dietary fiber, fats, proteins, sugars, vitamins, carotenoids, phenolics, lignans, volatile compounds, cyanogenic glycosides (amygdalin up to 4.9%)	Anticancer, anti-inflammatory, hepatoprotective	Treatment of gynecological, respiratory, and digestive disorders; anticancer signaling pathways	[112,119]
*Prunus* *africana* (Hook.f.) Kalkman	Phytosterols (1.5–2.5% of the dry weight of the bark), phenols (3 and 7 mg GAE/g extract), triterpenes (0.5–3.5% of the dry weight of the bark), fatty acids, and long-chain fatty alcohols	Anti-inflammatory, analgesic, antimicrobial, antioxidant, antiviral, antimutagenic, anti-asthmatic, antiandrogenic	Used for cancer treatment by limiting tumor growth and metastasis	[29,120]
*Prunus* *dulcis* (Mill) D.A. Webb	Almond seeds contain fixed oils (38.8%), phenolic compounds, minerals, vitamins	Anti-inflammatory, immunostimulant, antiproliferative	Treatment of IBS, constipation, and cancer; chemopreventive properties	[82]
*Prunus* *domestica* L.	Phenolic acids (gallic acid 0.81 µg/mg extract in plum native extract), flavonoids (quercetin 0.55 µg/mg extract in plum native extract), anthocyanins	Antioxidant, anticancer	Prevention of CRC; reduces oxidative damage, supports cancer drug synergism	[37,121]

Legend: GAE: gallic acid equivalents; QE: quercetin equivalents; CRC: colorectal cancer; IBS: irritable bowel syndrome; mg: milligrams; µg: micrograms; ng: nanograms.

**Table 2:** Anticancer perspectives of *Prunus* species along with their mechanisms of action and signaling pathways.

Scientific Name of the Plant	Cancer Type	Model	Mechanism of Action	Target	References
*Prunus domestica* L.	CC	Caco-2 cells	Decreases proinflammatory markers (NF-κB, Cox-2, iNOS), modulates AKT/mTOR and miRNA pathways	AKT/mTOR pathway, miR-143	[37]
*Prunus avium* L.	CC	Caco-2 cells	Influences the p38-MAPK signaling pathway	Cancer signaling pathways	[11,118]
*Prunus mume* Siebold and Zucc. extract	CRC	SW480, COLO, WiDr (in vitro); CRC model mice (in vivo)	Inhibits RelA, Bcl2, caspase 3; promotes Bax, cleaved caspase 3, and EGFR	RelA, Bcl2, EGFR	[45,80,127]
*Prunus amygdalus* L. var. *amara* (almond) oil	CC	Colo-320 and Colo-741 cells, in vivo animal studies	Decreases Ki-67 expression, caspase-independent apoptosis	Ki-67, caspases	[82,105]
*Prunus spinosa* L. ethanolic and aqueous extract	CRC	GLC, COLO320 cell lines	Cytotoxic effects, suppresses cancer growth	Colorectal cancer cells	[115]
*Prunus spinosa* L. extract (Trigno M)	CC, CRC	HCT116 cell line; colon cancer xenografts in mice	Delayed tumor progression and decreased tumor necrosis	Cancer signaling pathways	[53]
*Prunus domestica* L. methanolic extract	CC	Colon-26 cells, SW1116, HT29, Caco-2 cells	Significant growth inhibition and apoptosis induction	Cancer cell proteins, mitochondrial activity	[37]
*Prunus armeniaca* L. methanolic extract	CC	HCT-116 colon cells, Caco-2 cells	Inhibits growth in a dose-dependent manner, high antiproliferative activity	Cancer signaling pathways	[119]
*Prunus laurocerasus* L. methanolic extract	GC	AGS and MKN-45 cells	Induces significant cell death while preserving human fibroblasts	Cancer signaling pathways	[48]

Legend: NF-κB: nuclear factor kappa-light-chain-enhancer of activated B cells; Cox-2: cyclooxygenase-2; iNOS: inducible nitric oxide synthase; AKT/mTOR: protein kinase B (AKT)/mechanistic target of rapamycin (mTOR); miRNA: microRNA; p38-MAPK: p38 mitogen-activated protein kinase; CRC: colorectal cancer; Bcl2: B-cell lymphoma 2; Bax: proapoptotic factor; EGFR: epidermal growth factor receptor; Ki-67: cell proliferation marker.

## Data Availability

Data are contained within this article.
